# Rare Genetic Variation in 135 Families With Family History Suggestive of X-Linked Intellectual Disability

**DOI:** 10.3389/fgene.2019.00578

**Published:** 2019-06-26

**Authors:** Alba Sanchis-Juan, Christina Bitsara, Kay Yi Low, Keren J. Carss, Courtney E. French, Olivera Spasic-Boskovic, Joanna Jarvis, Michael Field, F. Lucy Raymond, Detelina Grozeva

**Affiliations:** ^1^Department of Haematology, NHS Blood and Transplant Centre, University of Cambridge, Cambridge, United Kingdom; ^2^Cambridge University Hospitals NHS Foundation Trust, NIHR BioResource, Cambridge, United Kingdom; ^3^Department of Medical Genetics, Cambridge Institute for Medical Research, University of Cambridge, Cambridge, United Kingdom; ^4^East Anglian Medical Genetics Service, Cambridge University Hospital, Cambridge, United Kingdom; ^5^Clinical Genetics Unit, Birmingham Women’s NHS Foundation Trust, Birmingham, United Kingdom; ^6^Genetics of Learning Disability Service (Hunter Genetics), Waratah, NSW, Australia

**Keywords:** intellectual disability, Mendelian disease, next-generation sequencing, autosomal dominant, X-linked, mosaicism

## Abstract

Families with multiple male children with intellectual disability (ID) are usually suspected of having disease due to a X-linked mode of inheritance and genetic studies focus on analysis of segregating variants in X-linked genes. However, the genetic cause of ID remains elusive in approximately 50% of affected individuals. Here, we report the analysis of next-generation sequencing data in 274 affected individuals from 135 families with a family history suggestive of X-linked ID. Genetic diagnoses were obtained for 19% (25/135) of the families, and 24% (33/135) had a variant of uncertain significance. In 12% of cases (16/135), the variants were not shared within the family, suggesting genetic heterogeneity and phenocopies are frequent. Of all the families with reportable variants (43%, 58/135), we observed that 55% (32/58) were in X-linked genes, but 38% (22/58) were in autosomal genes, while the remaining 7% (4/58) had multiple variants in genes with different modes on inheritance. This study highlights that in families with multiple affected males, X linkage should not be assumed, and both individuals should be considered, as different genetic etiologies are common in apparent familial cases.

## Introduction

Mendelian types of intellectual disability (ID) were first identified by documenting familial forms of the disease that had an X-linked mode of inheritance. These families were clinically recognized by an excess of affected males linked in a pedigree through mothers who were either mildly affected or unaffected. Initially, the excess of males with ID observed in the population was estimated to be due to a 10% contribution of X-linked disease genes to overall ID (Lehrke, 1972; Chelly and Mandel, 2001). Screening for single genes in affected individuals demonstrated that this was an overestimate (Mandel and Chelly, 2004; Raymond and Tarpey, 2006), and large-scale sequence analysis of the X chromosome in families demonstrated that X-linked variants do not always segregate in families thought previously to have X-linked disease (Raymond and Tarpey, 2006; Tarpey et al., 2009).

With the availability of next-generation sequence (NGS) analysis, the systematic identification of *de novo* mutagenesis through trio analysis, and the increasing recognition of autosomal recessive causes of neurodevelopmental disease, the opportunity arises to re-evaluate the mechanisms of disease in families with affected individuals ascertained with the clinical assumption of X-linked disease. To this end, we aimed to assess the contribution of genetic variation to disease in 274 individuals from 135 non-consanguineous families with suspected X-linked mode of inheritance. We studied families with multiple affected individuals with unexplained, moderate to severe non-syndromic ID using multiple NGS technologies.

## Materials and Methods

### Cohort

The criteria for selecting families for sequence analysis were the presence of at least two affected individuals in the family and no known genetic cause of disease previously identified through routine testing. The cohort consisted of DNA samples from 274 affected individuals from 135 families with moderate to severe non-syndromic ID and analyzed within the UK10K Rare Diseases project (Consortium et al., 2015) and/or the NIHR BioResource project (Ouwehand, 2019). Within the research ethical framework (IRAS 03/0/014 and 13/EE/0325), participants, parents, guardians, or consultee provided written informed consent to participate in the study.

**Figure 1:**
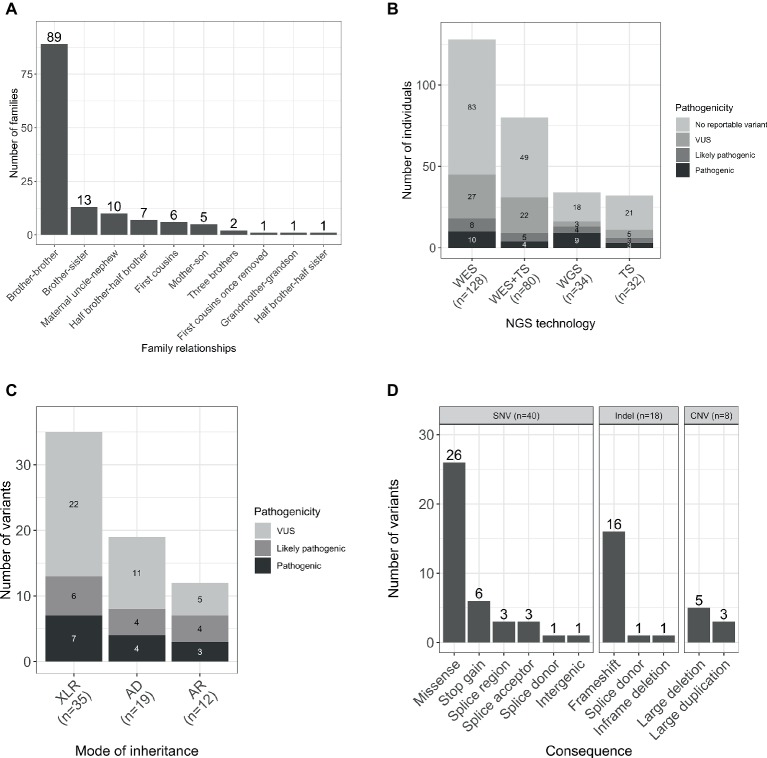
**(A)** Relationships within the studied families. **(B)** Number of individuals sequenced with each NGS technology by pathogenicity of the variant. **(C)** Total number of variants by mode of inheritance and pathogenicity scores (including SNVs/indels and CNVs). **(D)** Number of unique per family reported variants by type and consequence. WES, whole-exome sequencing; TS, targeted sequencing; WGS, whole-genome sequencing; AD, autosomal dominant; AR, autosomal recessive; XLR, X-linked recessive.

The gender ratio was 92% (252/274) male and 8% (22/274) female. The majority of the families were formed by two affected male individuals, and in four families, there were three affected individuals. The predominant relationship was brother-brother (66%, 89/135), but also maternal uncle-nephew (7%, 10/135), half-brothers (5%, 7/135), and first cousins (4%, 6/135), accounting for 83% (112/135) of the sample set. A smaller proportion was male-female including maternal grandmother-grandson, brother-sister, mother-son, and half-brother-half-sister (Figure 1A).

### Next-Generation Sequencing

Participants were sequenced using three different methodologies: whole-exome sequencing (WES), targeted sequencing, and whole-genome sequencing (WGS; Figure 1B).

WES was performed for 47% (128/274) participants, and candidate Single Nucleotide Variants (SNVs) and small insertions and deletions (indels) were identified as previously described (Consortium et al., 2015). Copy number variants (CNV) were called based on the WES data using CoNVex (Sifrim et al., 2016). Targeted sequencing of 565 genes associated with ID was performed for 12% (32/274) individuals. A full list of the sequenced genes and details of the experiment has been described elsewhere (Grozeva et al., 2015). Additionally, 29% (80/274) individuals were sequenced by both WES and targeted sequencing.

WGS analysis was performed for 12% (34/274) of the participants within the NIHR BioResource project, and SNVs/indels and structural variants were identified as previously described (Carss et al., 2017; Sanchis-Juan et al., 2018). Genome build GRCh37/hg19 was used for mapping and variant calling. Sequencing data have been deposited in EGA (accession numbers in Data Availability Statement) and all pathogenic variants in Supplementary Table S1 have been submitted to ClinVar (with accession numbers from SCV000897731 to SCV000897758).

### Variant Interpretation

Analysis of the variants obtained through the three different sequencing methodologies was largely similar. To identify pathogenic variants, a two-step protocol of automated variant filtering followed by manual review was used. In the automated filtering, variants were filtered by quality and frequency as previously described (Carss et al., 2017). SNV/indel analysis was restricted to known disease-associated genes, which were gathered from sources including OMIM^1^, DDG2P (Wright et al., 2015), and literature searches, then curated to ensure they comply with previously described criteria (Wright et al., 2015). The final list comprised of 1,334 genes. Subsequently, manual review of all the variants that passed the automated filtering in those genes was performed, according to the ACMG guidelines (Richards et al., 2015). Pathogenic, likely pathogenic and variants of uncertain significance (VUS) were reported to the recruiting clinician. All reported variants were independently confirmed.

## Results

### Variant Identification

A total number of 66 variants were reported in 38% (103/274) of individuals (Figure 1C). Seventy-six percent (50/66) of the variants were novel (not previously reported in HGMD Pro or ClinVar; Supplementary Table S1). Forty SNVs, 18 indels, and 8 CNVs provided 61, 27, and 12% of the reported variants, respectively. The CNVs were at known loci, previously reported to be associated with developmental delay, ID, or schizophrenia (Figure 1D). Coverage distribution for the eight CNVs and IGV plots for all the reported indels are available in Supplementary Figures S1, S2, respectively.

Reportable SNVs/indels were identified in 47 genes. The most frequent genes were *ATRX* and *SLC2A1*, with variants identified in four and three families, respectively, while other genes were only seen in a single family. This is consistent with the observed genetic heterogeneity of ID and the low contribution of variation at individual loci to the total prevalence (Carvill and Mefford, 2015; Grozeva et al., 2015).

### Correspondence Between Whole-Exome Sequencing and Targeted Sequencing Data

A total number of 80 individuals were sequenced with both WES and targeted sequencing platforms. Of these, 32 reportable variants were observed in 31 individuals. The correspondence between both platforms was assessed for SNVs/indels in genes included in the targeted sequencing gene panel, accounting for 19 variants.

The correspondence rate was 74% (14/19). Five variants were identified by WES but not targeted sequencing analysis. IGV plots for the alignments of these variants are in Supplementary Figure S3. Two of them were indels [NM_139058.2(*ARX*):c.1445_1448 + 1dup and NM_130839.2(*UBE3A*):c.983_987del] in a low coverage or poor quality region, supporting the view that there are still errors associated with indel calling from targeted sequencing compared to WES.

For the other three discordant variants, two were in regions with no read coverage in the targeted sequencing data [NM_000033.3(*ABCD1*):c.854G > A and NM_005629.3(*SLC6A8*):c.1693dup], and one had a read depth of one [NM_001493.2(*GDI1*):c.359C > T], therefore precluding variant detection. It has to be noted the presence of highly homologous pseudogenes and high GC content of *SLC6A8* genomic sequence that complicates the analysis of variants in this region (Yu et al., 2013) and could explain why the variant was missing from the targeted sequencing data.

As no individuals were sequenced by both WES and WGS, direct comparison of these technologies was not possible within this study. Furthermore, as only 34 samples received analysis by WGS in this study, in-depth analysis of coverage and yield from WGS compared to WES is not reported here (Carss et al., 2017).

### Diagnostic Yield in Families With a Family History Suggestive of X-Linked Intellectual Disability

**Figure 2:**
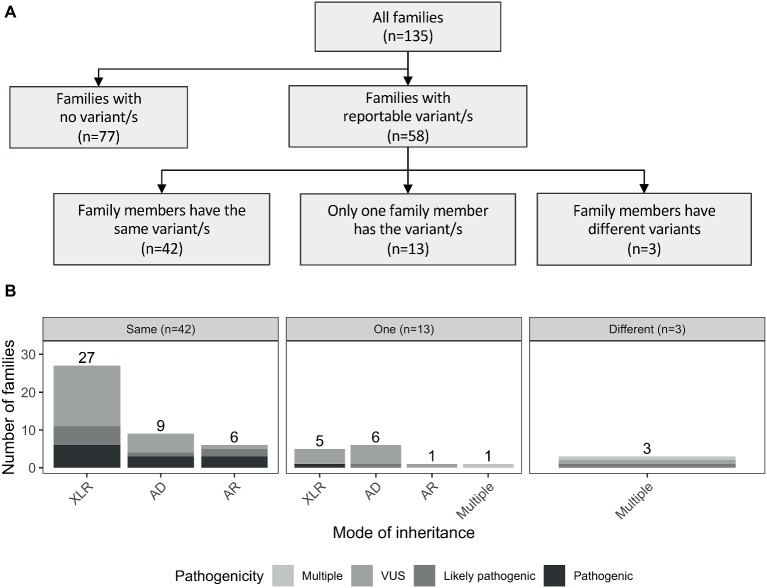
**(A)** Outline of the number of reported variants identified in the studied cohort. Families with reportable variants are separated in three categories: (1) number of families where affected members have the same variant (same); (2) number of families where only one family member has a variant (one); and (3) number of families where both affected individuals had different variants (different). **(B)** Number of families are showed by mode of inheritance of the variant/s. AD, autosomal dominant; AR, autosomal recessive; XLR, X-linked recessive.

Reportable variants were observed in 43% of the families (58/135): 19% (25/135) were pathogenic or likely pathogenic and 24% (33/135) were VUS. For families with multiple reportable variants presenting different pathogenicity assessments, the most deleterious one was considered. The reported variants were shared by individuals from the same family for the majority of cases (72%, 42/58). However, there were differences in 28% (16/58) of the families. More specifically, 13 had variants reported only for one individual, while in the remaining three families, both affected individuals had different reported variants (including one case where there was a shared and a unique variant only present in one individual; Figure 2A). The number of pathogenic variants by mode of inheritance of the gene is presented in Figure 2B.

In the 58 families with reportable variants, 55% (32/58) had variants in X-linked recessive genes, 38% (22/58) had variants in autosomal genes, and three additional families (5%) had multiple reportable variants presenting different modes of inheritance (Supplementary Table S2). Interestingly, these proportions also varied depending on the family structure. Brother-brother and maternal uncle-nephew relationships had a higher proportion of variants in X-linked genes [57 (21/37) and 86% (6/7), respectively] compared to variants in autosomal genes [41 (15/37) and 0% (0/37)]. However, for brother-sister pairs, the number of families with reported variants in autosomal genes (63%, 5/8) was higher than in X-linked genes (25%, 2/8).

Additionally, we note that in the brother-brother pairs, the proportion of variants in autosomal genes was higher for dominant (80%, 12/15) than recessive (20%, 3/15) modes of inheritance (Supplementary Figure S4). This was different in other relationships such as brother-sister, where only 20% (1/5) of the families had variants in autosomal dominant genes.

### Identification of Families With Variants in Autosomal Genes

As previously mentioned, 58 families had at least one reportable variant. As expected, a large number of these were in X-linked recessive genes (55%, 32/58; Figure 2B). Of those, the majority were novel, including a loss-of-function (LOF) variant in *HNRNPH2* gene, which was hemizygous in a maternal uncle and nephew (Family F133) that presented with severe ID, epilepsy, autism, developmental delay, and dysmorphic features. Pathogenic variants in this gene previously have only been reported in females, and so this case is the first report in the literature of a hemizygous LOF variant in *HNRNPH2* in males (additional information in Supplementary Data, Section 1.1).

Interestingly, a considerable proportion of families had reportable variants in autosomal genes (38%, 22/58), of which seven were in recessive genes, 15 were in dominant genes. Additionally, 7% of the families (4/58) presented with multiple variants with different modes of inheritance (Figure 2B). Three examples of families with variants in autosomal genes are presented below:

In family F002, we identified a frameshift variant in *TCF20* (chr22:42607507 GTC > G; NM_005650.2:c.3803_3804del; NP_005641.1: p.Arg168Thrfs*9), shared by two male siblings with severe ID (Torti et al., 2019). *De novo* and inherited pathogenic variants in *TCF20* have been recently linked to developing ID, dysmorphic features, hypotonia, and neurological impairments (Vetrini et al., 2019). Further investigations showed that another affected male sibling carried the same variant that was not observed in the mother and two unaffected male siblings by Sanger sequence analysis. The father was unavailable for segregation analysis. The parents were reported as phenotypically unaffected. Additional information with respect to this family and the variant is provided in Supplementary Data, Section 1.2.

In family F107, we identified a splice site variant in *SATB2* (chr2:200233432 T > C; NM_015265.3: c.598-2A > G) present in two male siblings (Bengani et al., 2017). Sanger sequencing analysis of peripheral blood-derived DNA from both parents revealed a normal sequence at this base. While most of the variants reported in *SATB2* are *de novo*, the observation that both siblings in this family shared the same LOF variant suggests that one of the parents was either gonadal mosaic or had too low level of somatic mosaicism to be detected by Sanger sequence analysis. Additional information is provided in Supplementary Data, Section 1.3.

In family F004, a shared 5 bp deletion frameshift variant was identified in *UBE3A* (chr15:25616333 CATTGT>C; NM_130839.2:c.983_987del; NP_570854.1: p.Tyr328Cysfs*18) in two affected male siblings. The children presented with global developmental delay with absent or minimal speech and significantly delayed age of walking. The two siblings had been previously tested for Angelman syndrome using microarray and methylation assays, which were both negative. As *UBE3A* is an imprinted gene, there could be four possible origins of the variant in the boys: (1) maternal germline mosaicism; (2) low level somatic mosaicism in the mother; (3) a paternally inherited variant in the mother; or (4) a *de novo* variant in the mother on the paternally inherited allele. The mother of the siblings was not available for further testing; therefore, the origin of this variant in the two boys could not be determined. Additional information is provided in the Supplementary Data, Section 1.4.

## Discussion

The aim of this study was to assess the contribution of genetic variation to disease in families affected with moderate to severe non-syndromic ID, with suspected X-linked inheritance due to family history. The overall diagnostic yield was 19% (25/135), and 24% (33/135) of the studied families had a VUS, comparable to those previously reported in similar studies (Tzschach et al., 2015; Hu et al., 2016). However, 57% (77/135) of the studied cohort remained unresolved. This could be because some of the studied individuals may have undetected pathogenic variants in coding exonic regions of low pull-down efficiency due to using targeted sequencing or WES. Others may harbor variants that were not called using the current algorithms that were filtered out during quality control filtering or were in genes that were absent from our curated gene list. Non-coding regions or complex rearrangements were also missed (Sanchis-Juan et al., 2018) in the families only tested using WES and/or targeted sequencing and oligogenic or multigenic modes of inheritance were not considered in any individuals (Niemi et al., 2018).

The studied cohort was ascertained due to the presence of moderate to severe non-syndromic ID and was relatively biased against recruitment of families with a clearly syndromic form of disease. Despite this, rare variants were observed in genes normally associated with distinct syndromic phenotypes [such as *MECP2* (MIM: 300673), *SPG7* (MIM: 607259), *MED12* (MIM: 305450), and *CASK* (MIM: 300749)] suggesting broader contribution of these genes to ID. Previous studies have also observed similar phenotypic variability in individuals with mutations in known syndromic ID-associated genes (Guerrini et al., 2000; Field et al., 2006; Hoyer et al., 2012; Rauch et al., 2012; Santen et al., 2013; Grozeva et al., 2015). Therefore, studying NGS data in large sample sets of patients with ID will help to provide information about the full spectrum of the associated phenotype presentation of mutations in a particular gene.

As expected, in families with reported variants, the majority of variants were shared in the affected individuals, but in 28% (16/58) of families, the variants were discordant. As ID is a heterogeneous disorder associated with many highly penetrant genes, the evidence of multiple different genetic etiologies within a family is perhaps unsurprising. In view of this, we recommend cautious use and interpretation of the ACMG guidelines, specifically BS4 category that is a strong predictor against pathogenicity when variants do not segregate with disease.

Furthermore, in 55% (32/58) of families with reported variants, these were identified, as expected, in genes on the X chromosome. Nevertheless, 38% of the families (22/58) presented variants in autosomal genes. Of these, only seven families had variants in autosomal recessive genes, consistent with previous results that have shown that ID is infrequently recessive in an outbred population (Grozeva et al., 2015; Martin et al., 2018).

Interestingly, 15 families had variants in autosomal genes that were shared by both members of the family, nine were in genes with a dominant mode of inheritance (4/9 were pathogenic and 5/9 were VUS; Figure 2B). In eight families, this occurred in brother-brother pairs, and in one family, this was observed in a mother-son pair (Supplementary Figure S4). Three of nine families are further described in Supplementary Data (Sections 1.2–1.4). No second pathogenic event was identified in any individual that had WES or WGS performed from these nine families, and in those that had targeted sequencing only, variants identified were likely or clearly pathogenic SNVs. Possible explanations for the observed familial variants in autosomal genes shared by the pairs are reduced penetrance of the variant in a parent, parent of origin effects for imprinted genes, germ line mosaicism in a parent, and low level somatic mosaicism in a parent that was undetectable. Mosaicism has been increasingly recognized as mutational mechanism in disease, with studies suggesting it may explain as much as 3.8–28.5% of apparently *de novo* variants (Biesecker and Spinner, 2013; Xu et al., 2015; Rahbari et al., 2016; Zillhardt et al., 2016; Jonsson et al., 2018).

This study uses NGS approaches in families affected with undiagnosed, moderate to severe, non-syndromic ID with a family history consistent with X-linked mode of inheritance. We show that, although X-linked inheritance is frequent in families with more than one affected individual, non-shared variants and autosomal inheritance are a significant cause of disease. Our results highlight the need to delineate X-linked disorders from other types of inheritance in order to clinically manage the condition in the family and to provide more accurate recurrence risk estimates. We therefore recommend more extensive genomic testing of families with potential X-linked mode of inheritance.

## Data Availability

UK10K sequencing data have been deposited in EGA under the name UK10K_RARE_FIND with accession EGAS00001000128. The variant calling data set is entitled UK10K_RARE_FIND REL-2013-10-31 (EGAD00001000750). Genomic data for the individuals sequenced by WGS are available at EGA with accession EGAD00001004522. All pathogenic variants in Supplementary Table S1 have been submitted to ClinVar, with accession numbers from SCV000897731 to SCV000897758 (www.ncbi.nlm.nih.gov/clinvar).

## Ethics Statement

Within the research ethical framework (IRAS 03/0/014 and 13/EE/0325), participants, parents, guardians, or consultee provided written informed consent to participate in the study.

## Author Contributions

DG and FR designed the study. DG, AS-J, CB, KL, and CF performed the formal analysis and investigation. KC, DG, AS-J, and OS-B performed data processing and curation. FR, JJ, MF, and clinicians within the GOLD Consortium recruited participants and collected the clinical data and samples. DG, AS-J, and FR wrote the manuscript. All authors read and approved the final manuscript.

### Conflict of Interest Statement

The authors declare that the research was conducted in the absence of any commercial or financial relationships that could be construed as a potential conflict of interest.

The reviewer CS declared a past co-authorship with one of the authors FR to the handling editor.

## Footnotes

^1^http://www.omim.org/

## Supplementary Material

SUPPLEMENTARY TABLE S1 List of participants and variants identified.

SUPPLEMENTARY TABLE S2 Number and proportion of families with reportable variants by mode of inheritance. XLR = X-linked recessive; auto = autosomal.

SUPPLEMENTARY FIGURE S1 Eight reported copy number variants (CNVs) identified by whole-exome sequencing (WES). Plots show changes in coverage of short-read WES across the entire chromosome (normalized depth t score using CNView, n=250) (Collins, 2016). Deletions and duplications are colored in red and blue respectively. SegDup=sites of multiallelic segmental duplications. All family relationships with reported CNVs are brother-brother, except from F130 (half-brothers) and F113 (mother-son). **(A)** Deletion in F007 identified in both affected individuals. **(B)** Duplication in F019 identified only in one affected individual. **(C)** Deletion in F031 identified only in one affected individual. **(D)** Duplication in F113 identified in both mother (mildly affected) and affected son. **(E)** Duplication in F115 identified at the nearly end of chromosome X in both affected individuals. **(F)** Deletion in F126 identified in both affected individuals. **(G)** Deletion in F129 identified in both affected individuals. **(H)** Deletion in F130 identified in one affected individual.

SUPPLEMENTARY FIGURE S2 Reportable insertions/deletions identified in this study. Plots were obtained using IGV for the families **(A)** F001, **(B)** F002, **(C)**, F004, **(D)** F005, **(E)** F006, **(F)** F009, **(G)** F011, **(H)** F012, **(I)** F013, **(J)** F025, **(K)** F093, **(L)** F108, **(M)** F110, **(N)** F114, **(O)** F118, **(P)** F125, **(Q)** F127, **(R)** F128.Additional details of the variants are present in **Supplemental Table 1**.

SUPPLEMENTARY FIGURE S3 Correspondence between WES and targeted sequencing data. IGV plots for the variants identified by WES (bottom) but absent from the targeted sequencing data (top) in the individuals **(A)** F001-A, **(B)** F004-A, (C) F114-A, **(D)** F109-B and **(E)** F112-A.

SUPPLEMENTARY FIGURE S4 **(A)** Number of families with reported variants identified in the studied cohort by mode of inheritance. If multiple genes with different mode of inheritance are identified in a family, the most deleterious pathogenicity is considered. **(B)** Number of families are grouped if the variants are: shared by all the family members (Same), only present in one individual (One) or multiple family members have different reportable variants (Different). Shading represents the mode of inheritance of the variant/s. AD=autosomal dominant; AR=autosomal recessive; XLR=X-linked recessive.
