# Supplementary material for: Rare Genetic Variation in 135 Families With Family History Suggestive of X-Linked Intellectual Disability
Source: Front Genet. 2019 Jun 26;10:578. doi: 10.3389/fgene.2019.00578 (PMC6609311; doi:10.3389/fgene.2019.00578)
Supplement: SUPPLEMENTARY FIGURE S1 — Eight reported copy number variants (CNVs) identified by whole-exome sequencing (WES). Plots show changes in coverage of short-read WES across the entire chromosome (normalized depth t score using CNView, n=250) (Collins, 2016). Deletions and duplications are colored in red and blue respectively. SegDup=sites of multiallelic segmental duplications. All family relationships with reported CNVs are brother-brother, except from F130 (half-brothers) and F113 (mother-son). (A) Deletion in F007 identified in both affected individuals. (B) Duplication in F019 identified only in one affected individual. (C) Deletion in F031 identified only in one affected individual. (D) Duplication in F113 identified in both mother (mildly affected) and affected son. (E) Duplication in F115 identified at the nearly end of chromosome X in both affected individuals. (F) Deletion in F126 identified in both affected individuals. (G) Deletion in F129 identified in both affected individuals. (H) Deletion in F130 identified in one affected individual. [file Data_Sheet_1.PDF]

Supplementary Material

Rare Genetic Variation in 135 Families with Family History Suggestive of X-linked Intellectual Disability

Alba Sanchis-Juan[1,2], Christina Bitsara[3], Kay Yi Low[3], Keren J. Carss[1,2], Courtney E. French[2,3], Olivera Spasic-Boskovic[4], Joanna Jarvis[5], Michael Field[6], F. Lucy Raymond[2,3*], Detelina Grozeva[3]

[1]Department of Haematology, University of Cambridge, NHS Blood and Transplant Centre, Cambridge CB2 0PT, UK

[2]NIHR BioResource, Cambridge University Hospitals NHS Foundation Trust, Cambridge Biomedical Campus, Cambridge CB2 0QQ, UK

[3]Department of Medical Genetics, Cambridge Institute for Medical Research, University of Cambridge, Cambridge, CB2 0XY, UK

[4]East Anglian Medical Genetics Service, Cambridge University Hospital, Cambridge, CB2 0QQ, UK

[5]Clinical Genetics Unit, Birmingham Women's NHS Foundation Trust, Metchley Park Road, Edgbaston, Birmingham, B15 2TG, UK

[6]Genetics of Learning Disability Service, Hunter Genetics, Waratah, New South Wales 2298, Australia

*** Correspondence:**

Professor F. Lucy Raymond

flr24@cam.ac.uk

Contents

1. Supplementary Data 3

1.1 Family F133: Loss-of-function variant in *HNRNPH2* 3

1.2 Family F002: Loss-of-function variant in *TCF20* 5

1.3 Family F107: Loss-of-function variant in *SATB2* 7

1.4 Family F004: Loss-of-function variant in *UBE3A* 9

2. Supplementary Figures 11

2.1 Figure S1 11

2.2. Figure S2 14

2.3 Figure S3 20

2.4 Figure S4 22

3. References 23

1. Supplementary Data

1.1 Family F133: Loss-of-function variant in *HNRNPH2*

In Family F133, the phenotype of the affected male individuals included severe ID, epilepsy, autism, developmental delay, dysmorphic and coarse facial features, absent speech, microcephaly (9-25th percentile), short stature, underweight (0.4th percentile), aggressive and self-harming behavior which is consistent with the previously described affected individuals (Bain et al., 2016). All the affected males in the family have very similar presentation of disease.

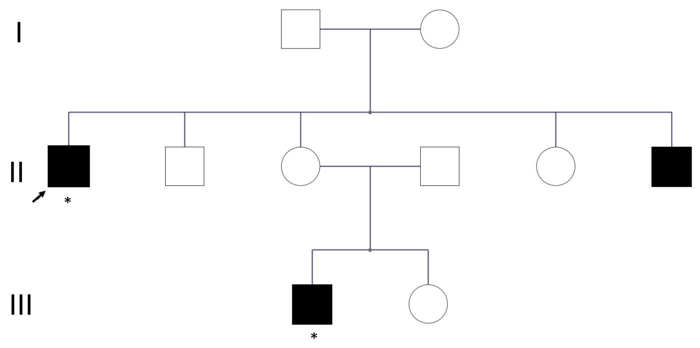

Pedigree structure of Family F133

A hemizygous loss-of-function (LOF) variant was observed in *HNRNPH2*:

ChrX(GRCh37/hg19):g.100668108 G>T

NM_001032393:c.1132G>T

NP_001027565:pGlu378*

This variant was at the 3' end of *HNRNPH2* and was present in both male affected individuals (maternal uncle and nephew). Recurrent missense mutations in this gene have been described for intellectual disability (ID) in females (Bain et al., 2016). Currently no LOF variants in gnomAD or disease cohorts have been reported to date. We note that this variant is in the last exon of the protein and is likely to escape nonsense-mediate decay (NMD). This variant was therefore reported as a variant of uncertain significance. No other candidate variants were identified in either of the uncle and proband in whom Whole-Exome Sequencing (WES) was performed.

The Integrated Genomics Viewer (IGV) (Robinson et al., 2011) plots show high-throughput sequencing reads in both affected individuals sequenced in this study:

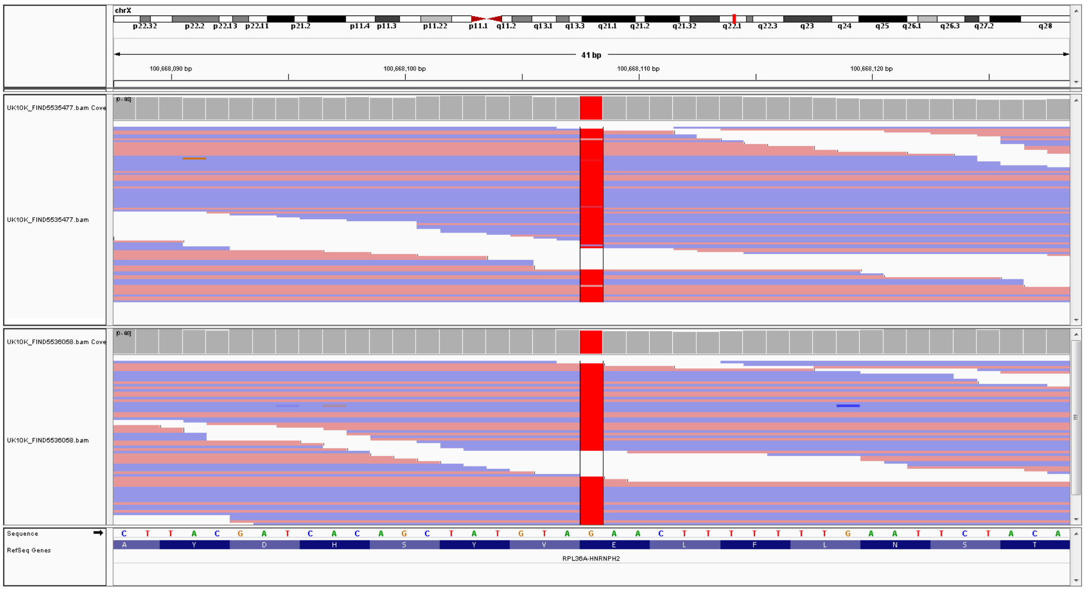

IGV plot of variant in *HNRNPH2* in Family F133

1.2 Family F002: Loss-of-function variant in *TCF20*

An extensive summary of the clinical features associated with variants in *TCF20* has been recently published (Torti et al., 2019). This paper also includes the family presented here (F002) under the corresponding ID "Family 17".

The three siblings sharing the variant (two included in this project) have ID, autism spectrum disorder, other neurobehavioral and related concerns (anxiety, depression, obsessive/perseverating behaviors and no recognizable facial gestalt). The three adult brothers live in supported accommodation, requiring assistance with self-care and work in a supported employment.

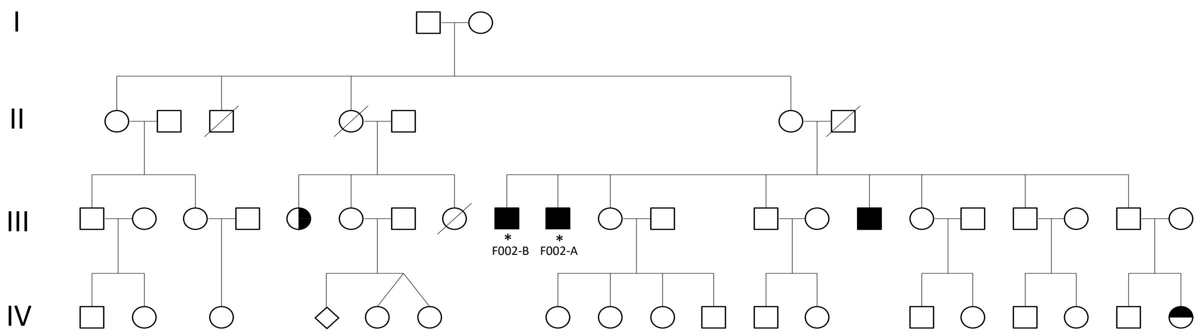

Pedigree structure of Family F002

The following frameshift variant was observed in family F002 in *TCF20*:

Chr22(GRCh37/hg19):g. 42607507 GTC>G

NM_005650.2:c.3803_3804del

NP_005641.1: p.Arg168Thrfs*9

Manual investigation of the alignment using IGV showed that for the sibling sequenced by targeted sequencing (top panel), the region was not covered, but was covered in whole exome sequencing data from the brother (bottom panel). Further investigations confirmed the variant was present in both affected individuals and extensive co-segregated was recommended.

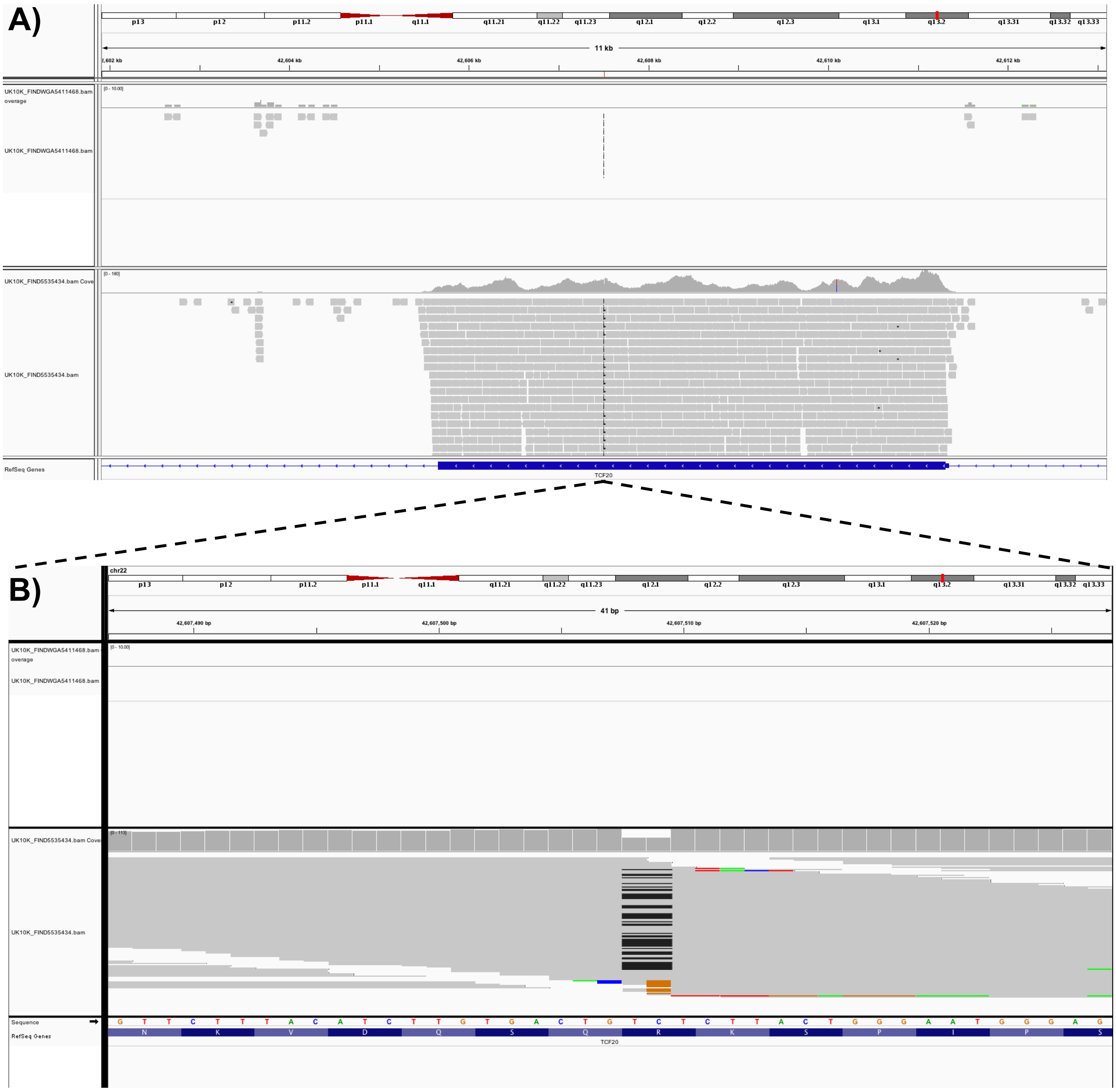

IGV plot of variant in *TCF20* in Family F002. Top panel corresponds with the targeted sequencing, and bottom panel is the WES. A) Zoomed out representation of the region where the variant is. B) Pathogenic LOF variant in *TCF20,* only observed in one of the siblings from the sequencing data.

1.3 Family F107: Loss-of-function variant in *SATB2*

Extensive summary of the clinical features associated with variants in *SATB2* has been recently published (Bengani et al., 2017). This paper also included the family presented here (F107) with the corresponding IDs for the two brothers “29089” and “29090”.

In this family, formed by two affected brothers, targeted sequencing was performed for one individual, whilst the other had WES analysis. Common phenotypes in both include moderate/severe ID and developmental delay. Additionally, one of the brothers have lipodystrophy appearance and raised plasma alkaline phosphatase, while for the other sibling’s brain MRI showed prom cisterna magna, mild vermis hypoplasia and perisylvian syndrome.

An essential splice site variant in *SATB2* was observed in both individuals:

Chr2(GRCh37/hg19):g. 200233432 T>C

NM_015265.3: c.598-2A>G

The family structure and the corresponding chromatographs for family F107 are shown below (Figure S5). The affected boys (II:2 29089 and II:4 29090) share the same essential splice site mutation, which is absent in both parents (I:1 and I:2), suggesting gonadal mosaicism.

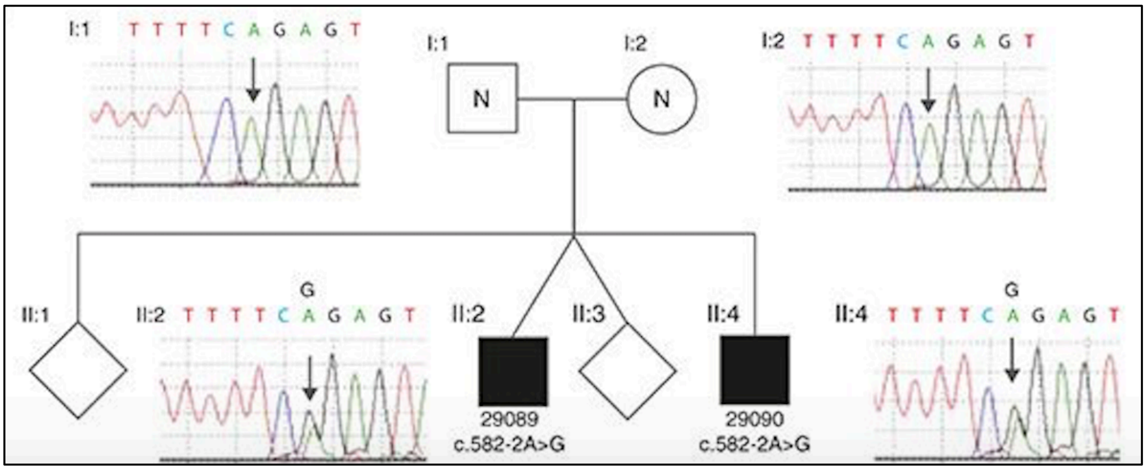

Pedigree structure of Family F107 and Sanger sequencing confirmations of the variant in *SATB2*. From Bengani et al., 2017.

IGV plots show reads supporting the alternate allele in both affected individuals.

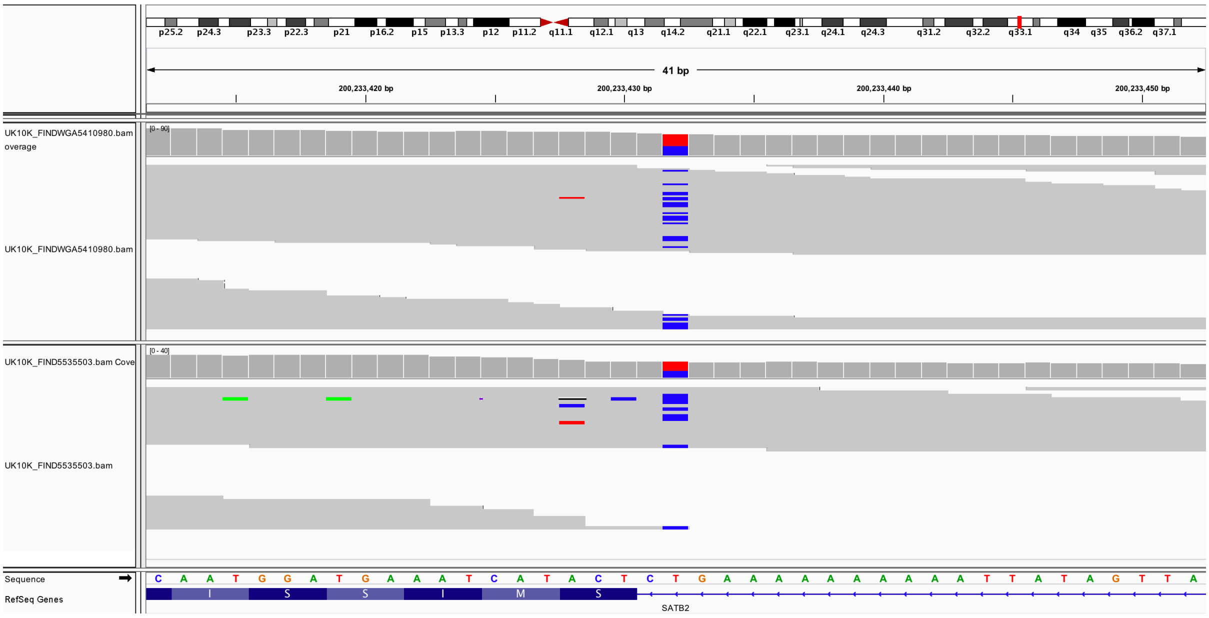

IGV plot of variant in *SATB2* in Family F107

1.4 Family F004: Loss-of-function variant in *UBE3A*

There are two affected brothers in the family. The disease presentation of the first brother involved global developmental delay with speech severely affected, dysmorphic features, microcephaly (<3rd percentile at 8 years of age), short attention span, well-behaved and normal height. Surgery for squint and amblyopia had been performed. The second brother presented with global developmental delay with mainly motor delay, microcephaly, hepatomegaly during first years of life, no speech, poor mobility, walked supported at age 4, progressed to walking independently at a later stage and alternating divergent squint. Height is at the 8-25th percentiles. Both of the siblings had normal MRI. Angelman syndrome had been suggested but routine testing for a microdeletion and abnormal methylation pattern was negative. The pedigree structure is represented in Figure S7:

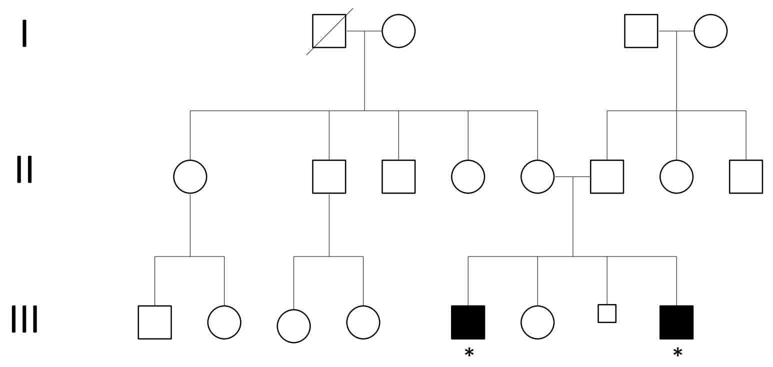

Pedigree structure of Family F004

A frameshift variant in *UBE3A* was observed in F004:

Chr15(GRCh37/hg19):g. 25616333 CATTGT>C

NM_130839.2:c.983_987del

NP_570854.1: p.Tyr328Cysfs*18

The IGV plots show high-throughput sequencing reads in both affected individuals:

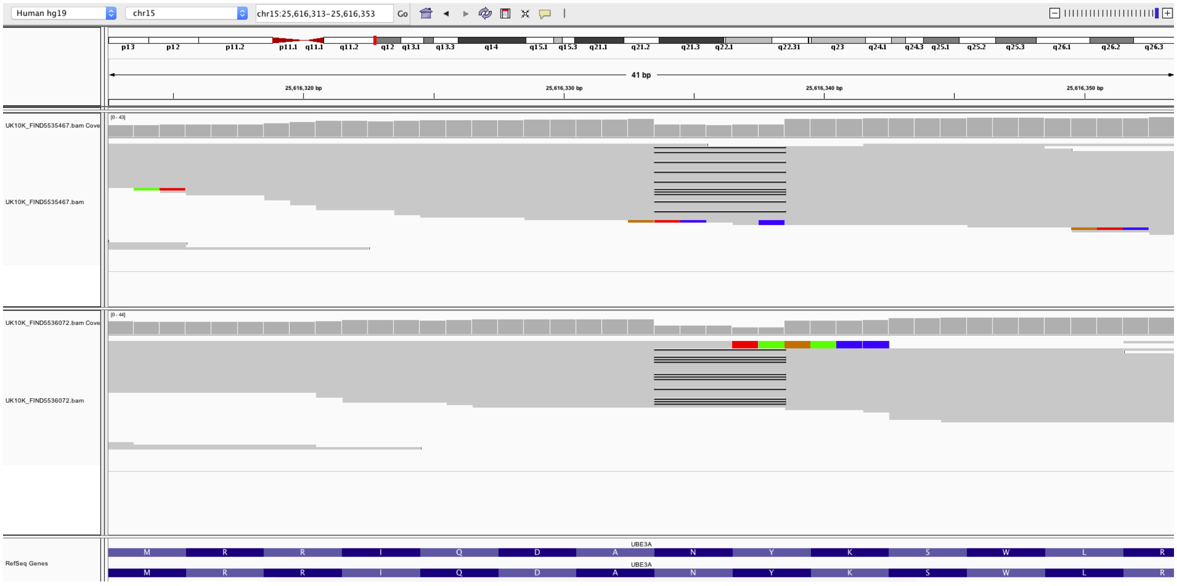

IGV plot of variant in *UBE3A* in Family F104

2. Supplementary Figures

2.1 Figure S1

Figure S1. Eight reported copy number variants (CNVs) identified by whole-exome sequencing (WES). Plots show changes in coverage of short-read WES across the entire chromosome (normalized depth t score using CNView, n=250) (Collins, 2016). Deletions and duplications are colored in red and blue respectively. SegDup=sites of multiallelic segmental duplications. All family relationships with reported CNVs are brother-brother, except from F130 (half-brothers) and F113 (mother-son). A) Deletion in F007 identified in both affected individuals. B) Duplication in F019 identified only in one affected individual. C) Deletion in F031 identified only in one affected individual. D) Duplication in F113 identified in both mother (mildly affected) and affected son. E) Duplication in F115 identified at the nearly end of chromosome X in both affected individuals. F) Deletion in F126 identified in both affected individuals. G) Deletion in F129 identified in both affected individuals. H) Deletion in F130 identified in one affected individual.

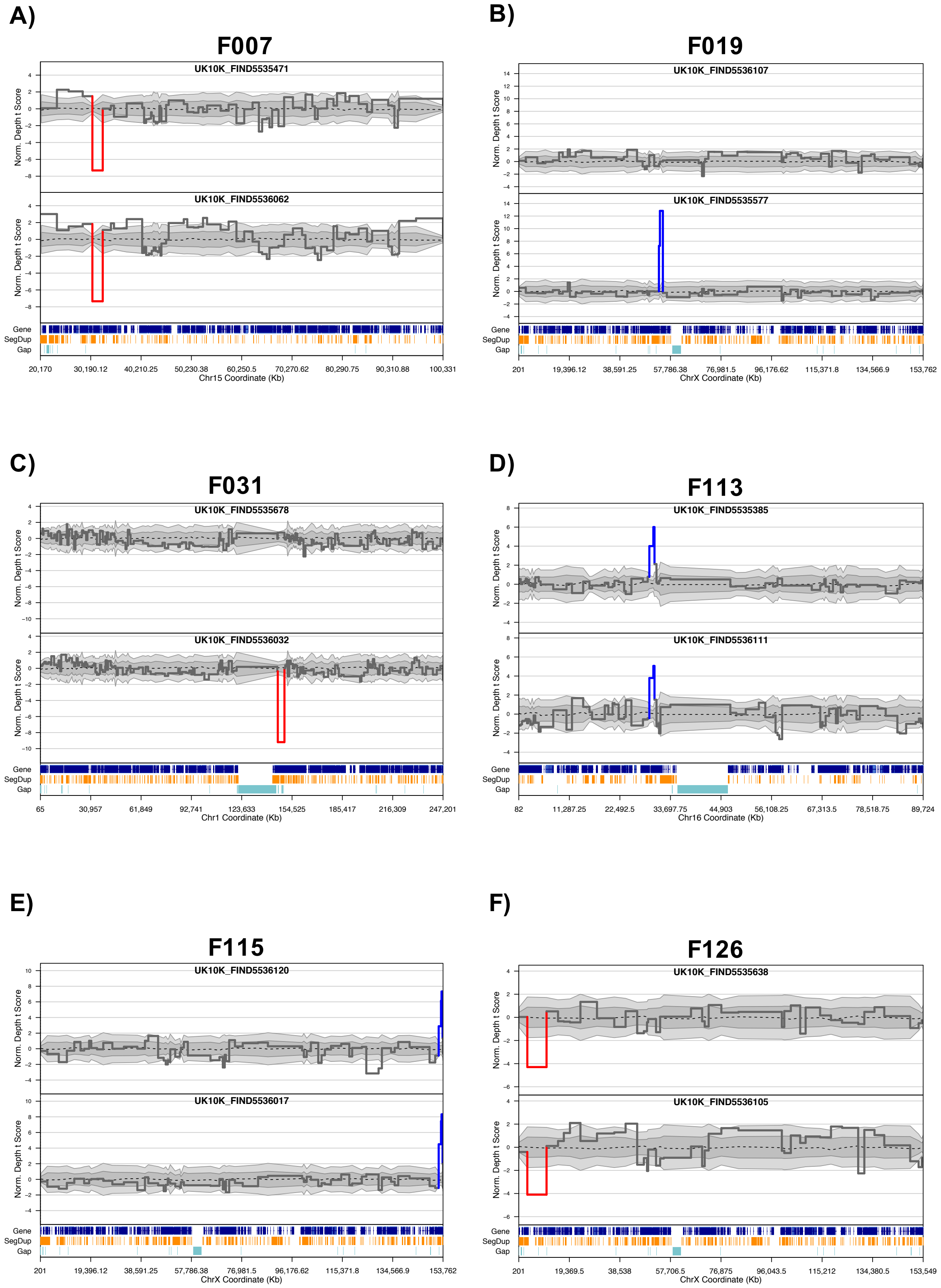
A)
F007
UK10K_FIND5535471
UK10K_FIND5536062
Norm. Depth t Score
Gene
SegDup
Gap
20,170
30,190.12
40,210.25
50,230.38
60,250.5
70,270.62
80,290.75
90,310.88
100,331
Chr15 Coordinate (Kb)
B)
F019
UK10K_FIND5536107
UK10K_FIND5535577
201
19,396.12
38,591.25
57,786.38
76,981.5
96,176.62
115,371.8
134,566.9
153,762
ChrX Coordinate (Kb)
C)
F031
UK10K_FIND5535678
UK10K_FIND5536032
65
30,957
61,849
92,741
123,633
154,525
185,417
216,309
247,201
Chr1 Coordinate (Kb)
D)
F113
UK10K_FIND5535385
UK10K_FIND5536111
82
11,287.25
22,492.5
33,697.75
44,903
56,108.25
67,313.5
78,518.75
89,724
Chr16 Coordinate (Kb)
E)
F115
UK10K_FIND5536120
UK10K_FIND5536017
201
19,396.12
38,591.25
57,786.38
76,981.5
96,176.62
115,371.8
134,566.9
153,762
ChrX Coordinate (Kb)
F)
F126
UK10K_FIND5535638
UK10K_FIND5536105
201
19,369.5
38,538
57,706.5
76,875
96,043.5
115,212
134,380.5
153,549
ChrX Coordinate (Kb)

G)

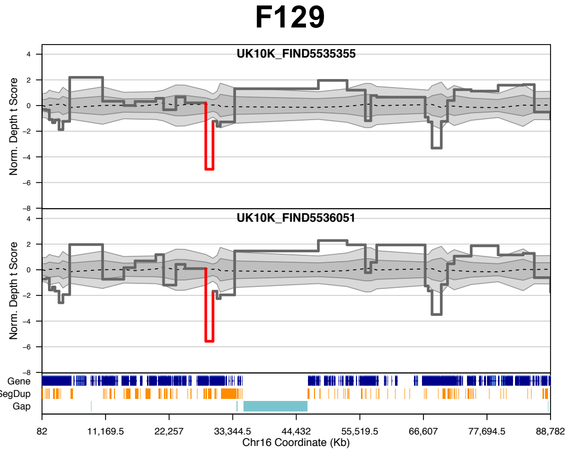

F129
UK10K_FIND5535355
UK10K_FIND5536051
Norm. Depth t Score
Gene
SegDup
Gap
82
11,169.5
22,257
33,344.5
44,432
55,519.5
66,607
77,694.5
88,782
Chr16 Coordinate (Kb)

H)

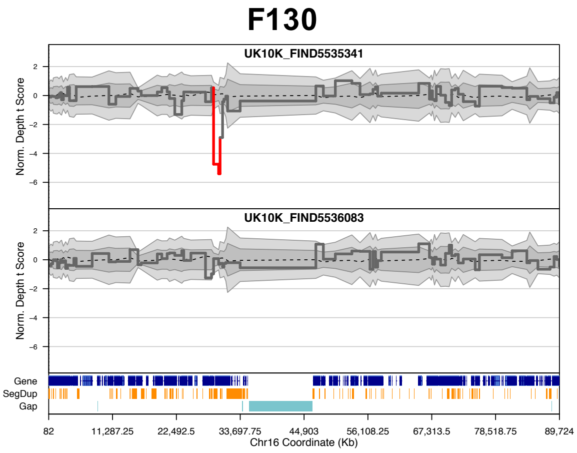

F130
UK10K_FIND5535341
UK10K_FIND5536083
Norm. Depth t Score
Gene
SegDup
Gap
82
11,287.25
22,492.5
33,697.75
44,903
56,108.25
67,313.5
78,518.75
89,724
Chr16 Coordinate (Kb)

2.2. Figure S2

Figure S2. Reportable insertions/deletions identified in this study. Plots were obtained using IGV for the families A) F001, B) F002, C), F004, D) F005, E) F006, F) F009, G) F011, H) F012, I) F013, J) F025, K) F093, L) F108, M) F110, N) F114, O) F118, P) F125, Q) F127, R) F128. Additional details of the variants are present in Supplemental Table 1.

A) F001: *ARX*, chrX:g.25025226A>ACCTG; NM_139058.2:c.1445_1448+1dup

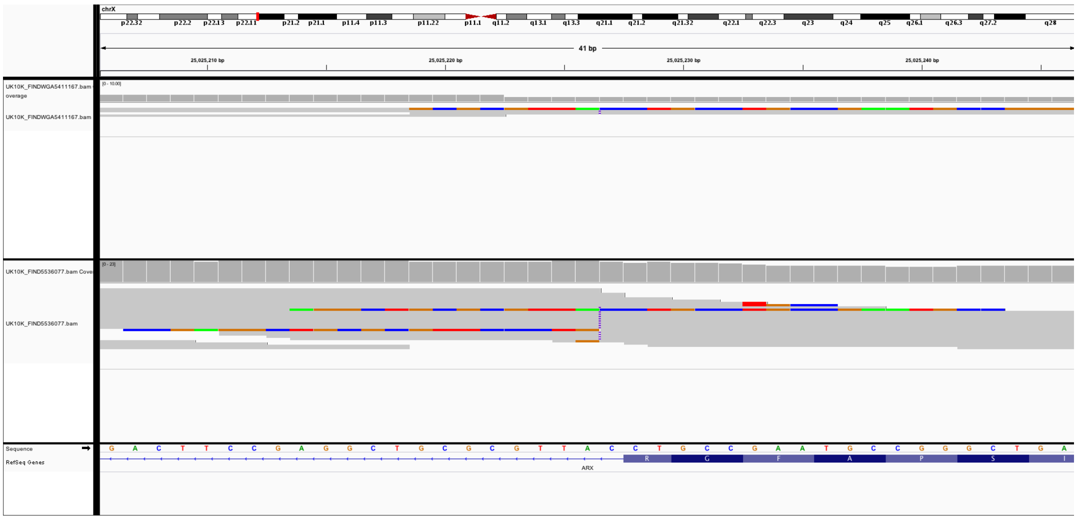

B) F002: *TCF20*, Chr22:g.42607507 GTC>G; NM_005650.2:c.3803_3804delGA
See Supplemental Data, section 1.2.

C) F004: *UBE3A*, chr15:g.25616333 CATTGT>C; NM_130839.2:c.983_987del

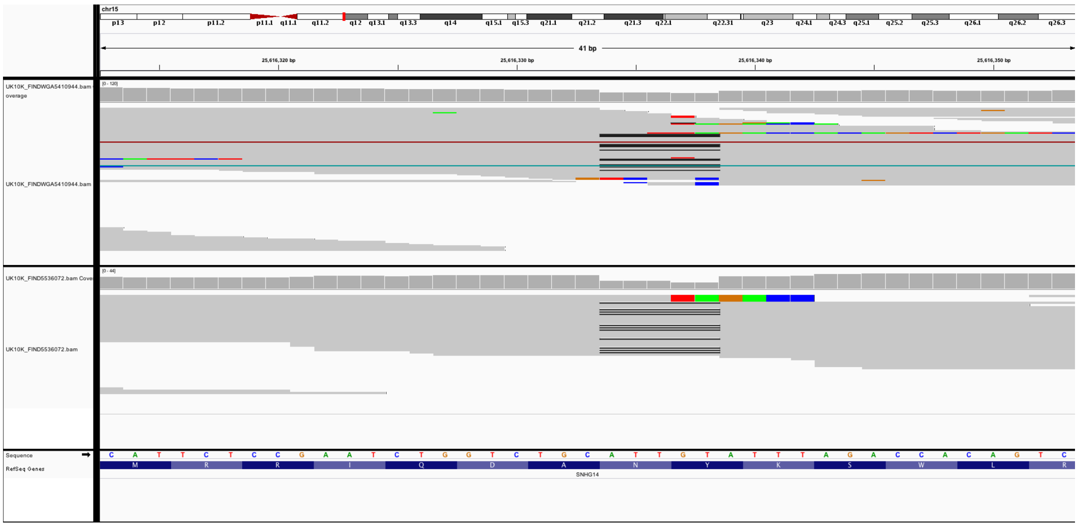

D) F005: *DLG3*, chrX:g.69674129 CCAGT>C; NM_021120.3:c.1369_1372del

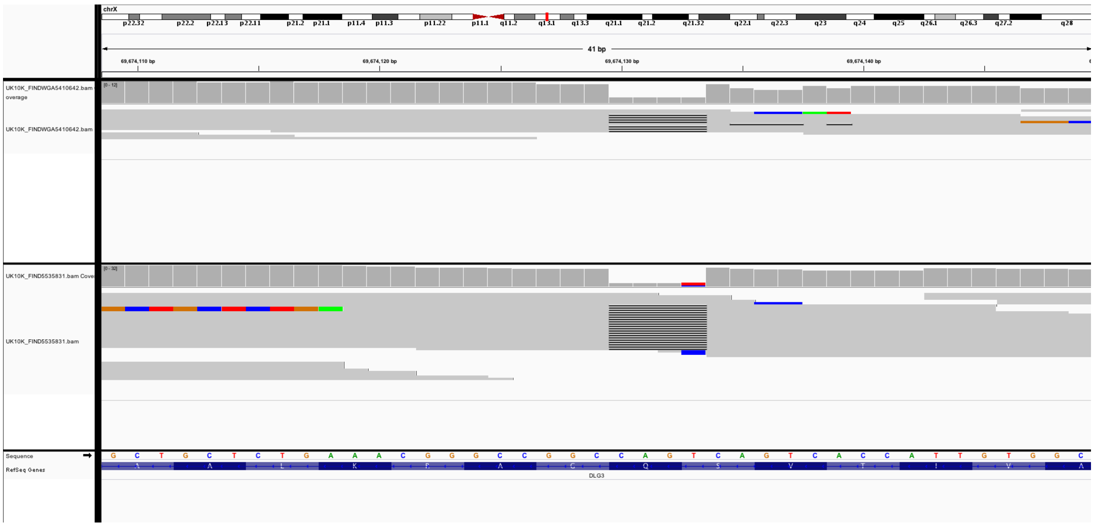

E) F006: *BRWD3*, chrX:g.79945282 AC>A; NM_153252.4:c.3791del

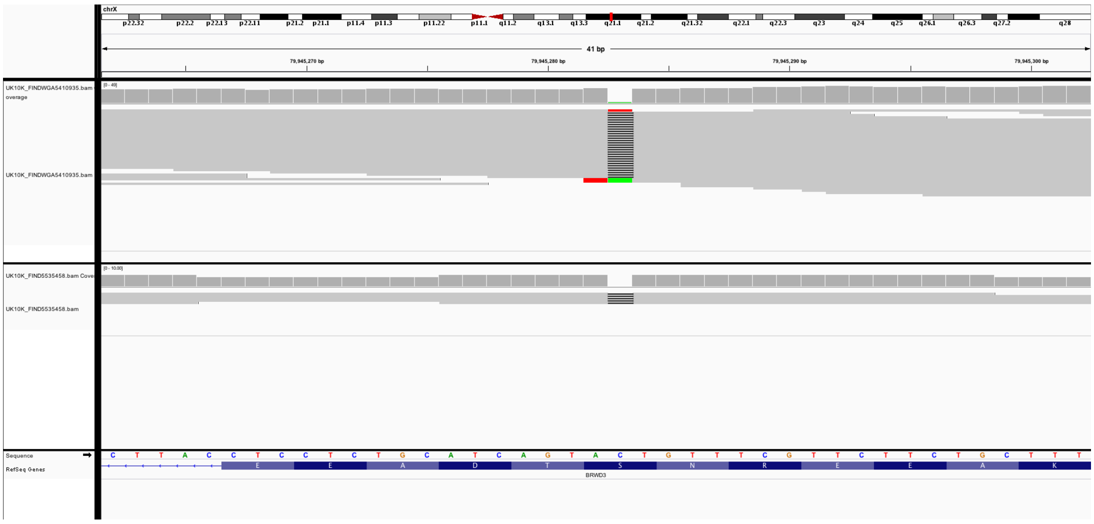

F) F009: *USP27X*, chrX:g. 49645215 TGA>T; NM_001145073.1:c.310_311del

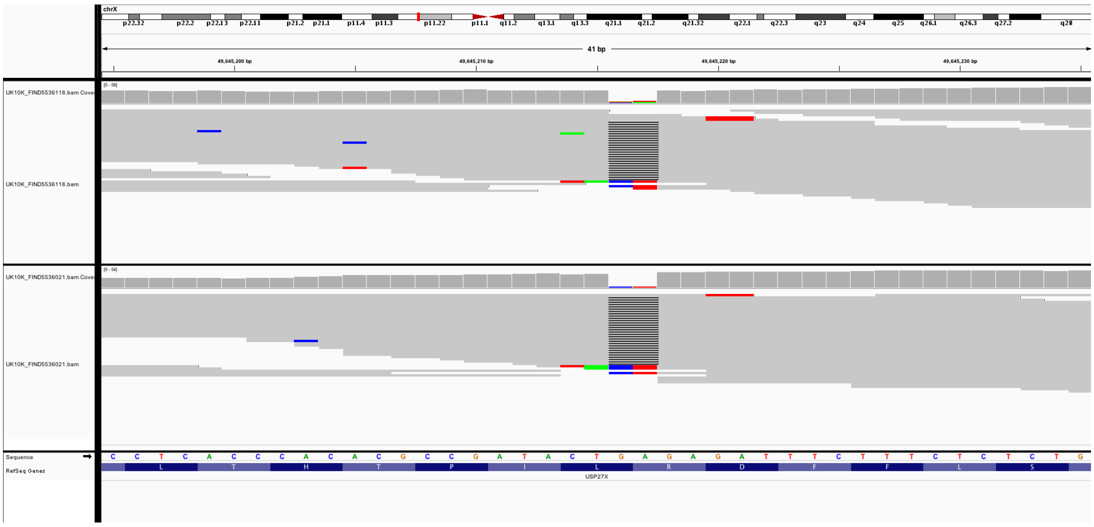

G) F011: *SPG7*, chr16:g.89620252 AAGC>A; NM_003119.2:c.1990_1992delCAG

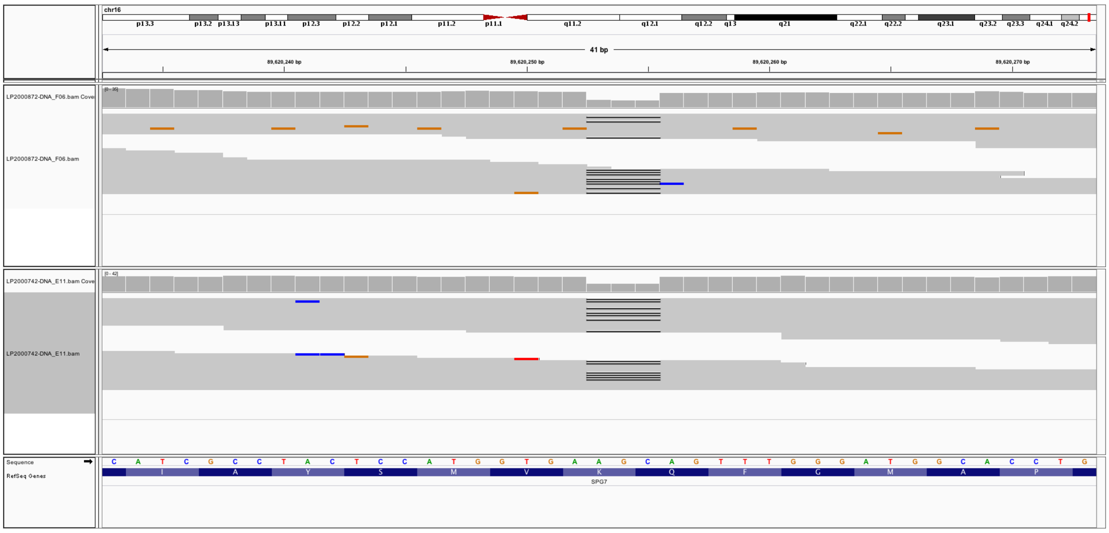

H) F012: *GJC2*, chr1:g.228345565 AT>A; NM_020435.3:c.107delT

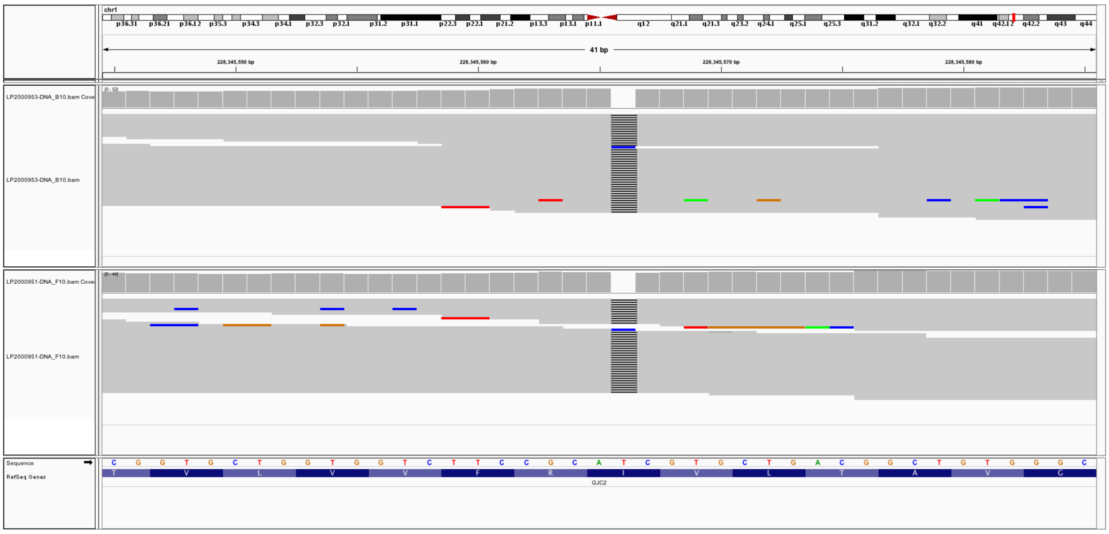

I) F013: *KIF1C*, chr17:g.4908177 CTGCAA>C NM_006612.5:c.1051_1055delAATGC

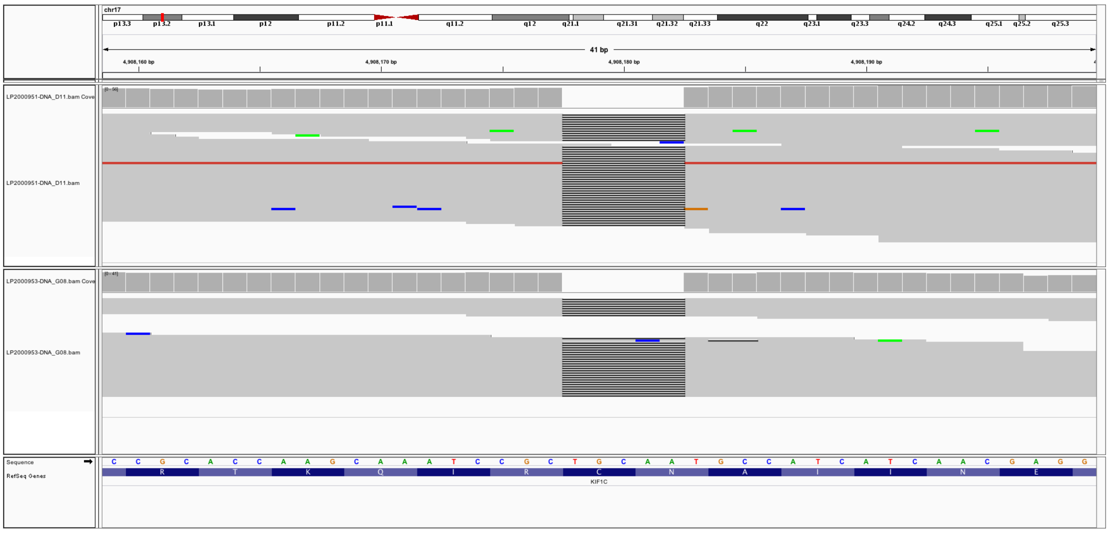

J) F025: *POGZ*, chr1:g.151403189 CAT>C; NM_015100.2:c.410_411del

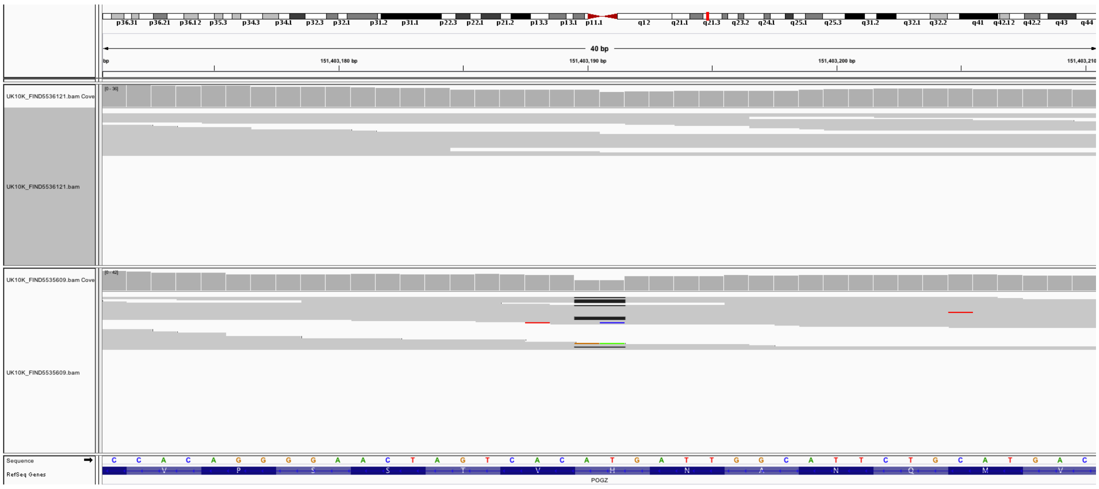

K) F093: *MPZ*, chr1:g.161276216 CG>C; NM_000530.6:c.486del

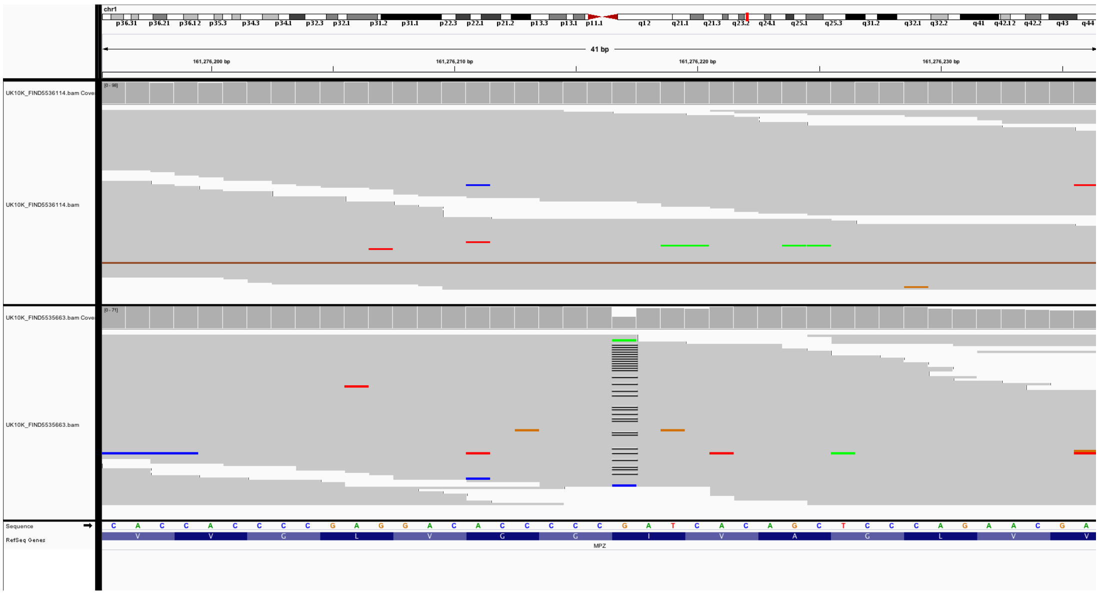

L) F108: *PQBP1*, chrX:g.48759667 CAGAG>C; NM_001032381.1:c.459_462del

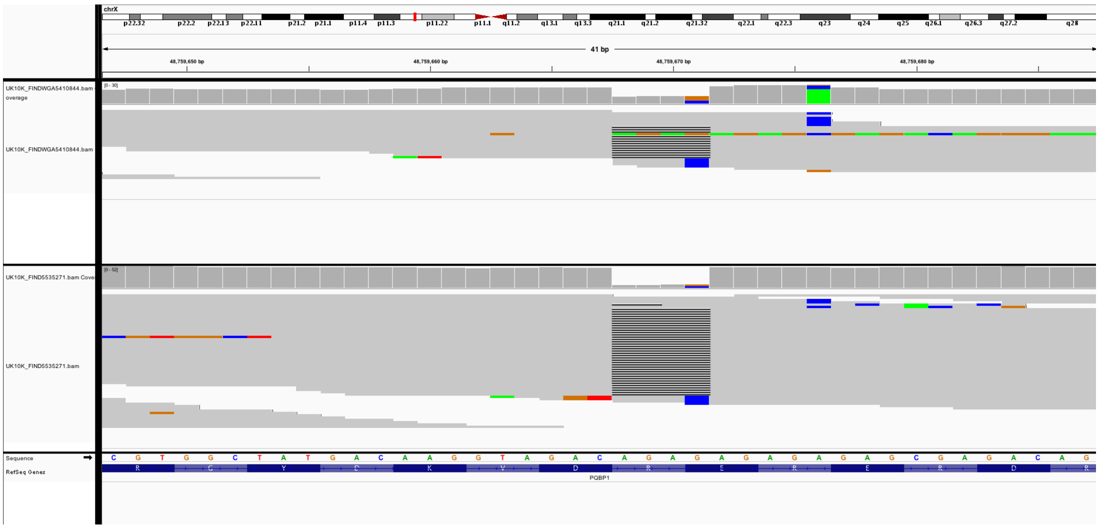

M) F110: *FRMPD4*, chrX:g.12735806 CG>C; NM_014728.2:c.2862del

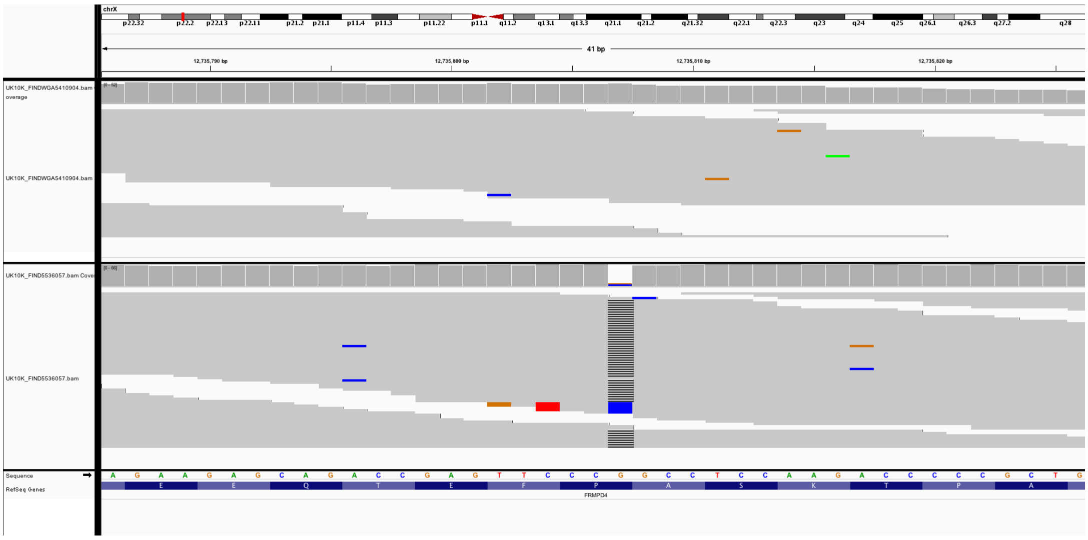

N) F114: *SLC6A8*, chrX:g.152960269 C>CG; NM_005629.3:c.1693dup

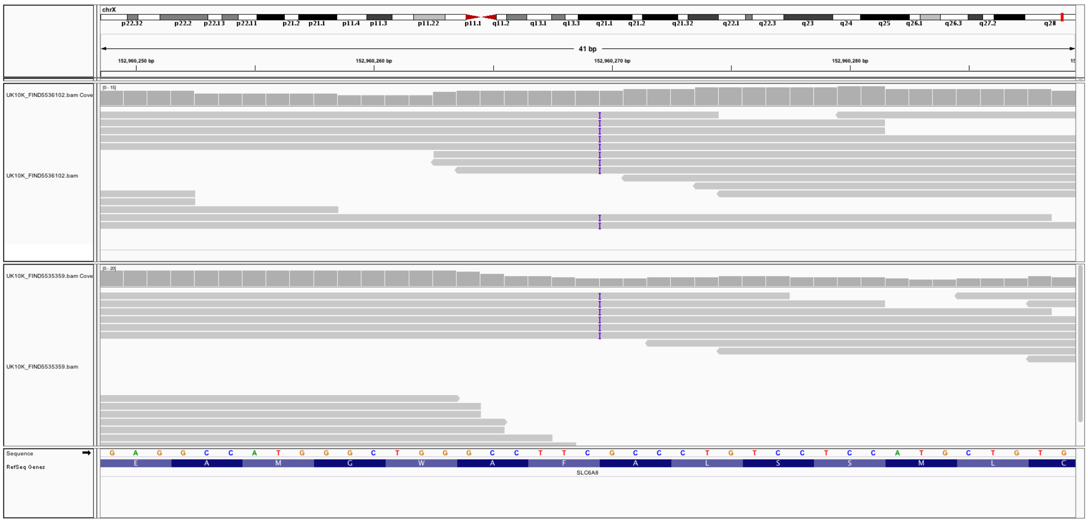

O) F118: *SLC6A8*, chrX:g.152959667 CTG>C; NM_005629.3:c.1340_1341del

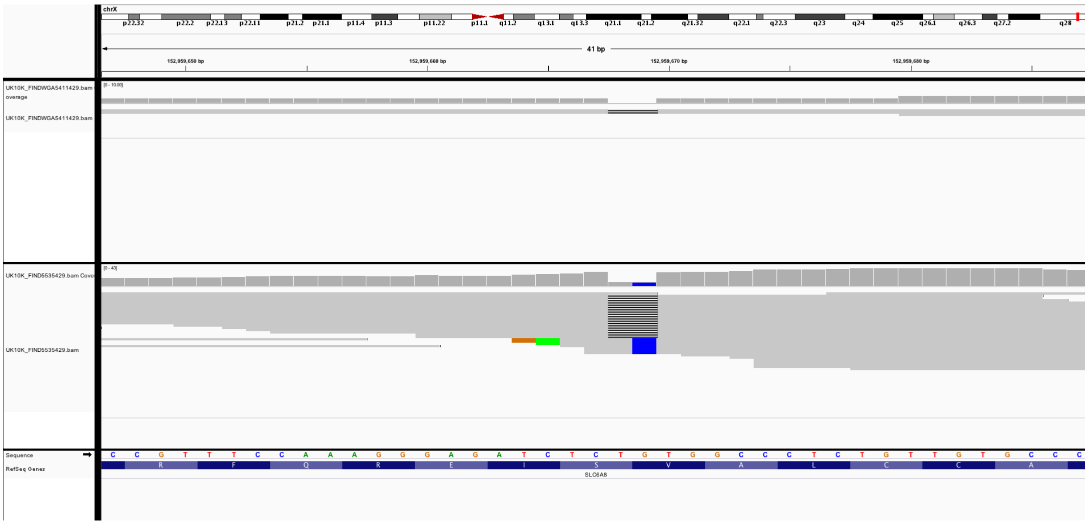

P) F125: *SRCAP*, chr16:g.30750696 CACTA>C; NM_006662.2:c.9338_9341del

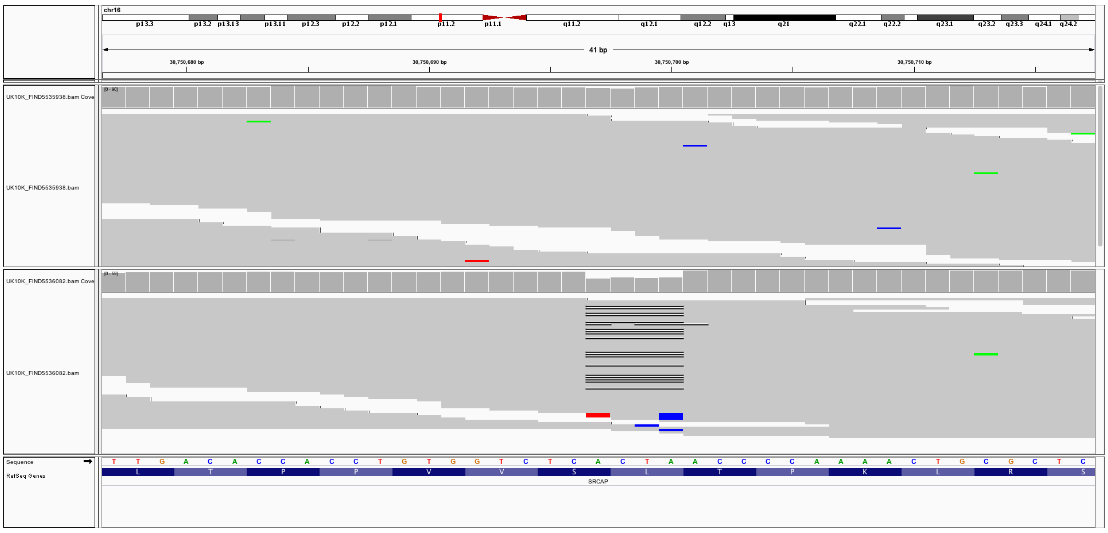

Q) F127: *FOXP2*, chr7:g.114271631 GT>G; NM_148899.3:c.647del

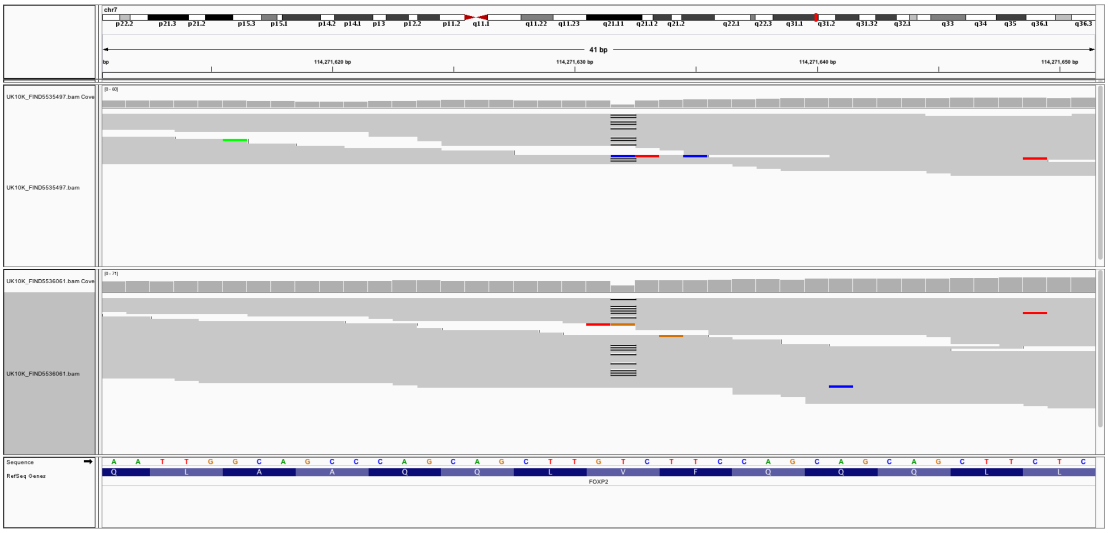

R) F128: *ITPR1*, chr3:g.4711413 AG>A; NM_002222.4:c.1918del

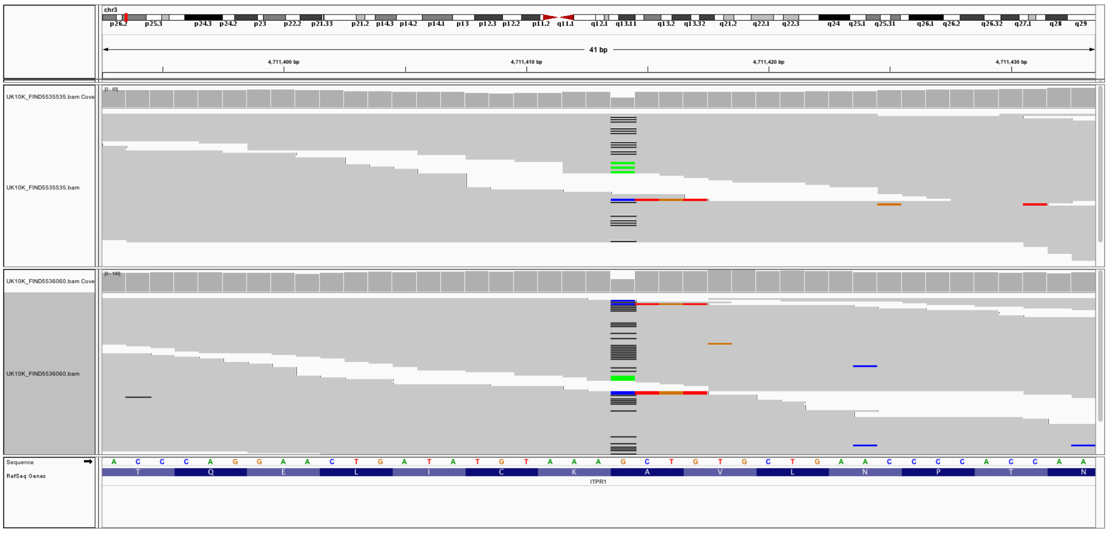

2.3 Figure S3

Figure S3. Correspondence between WES and targeted sequencing data. IGV plots for the variants identified by WES (bottom) but absent from the targeted sequencing data (top) in the individuals A) F001-A, B) F004-A, C) F114-A, D) F109-B and E) F112-A.

A) F001-A: *ARX*, chrX:g.25025226A>ACCTG; NM_139058.2:c.1445_1448+1dup

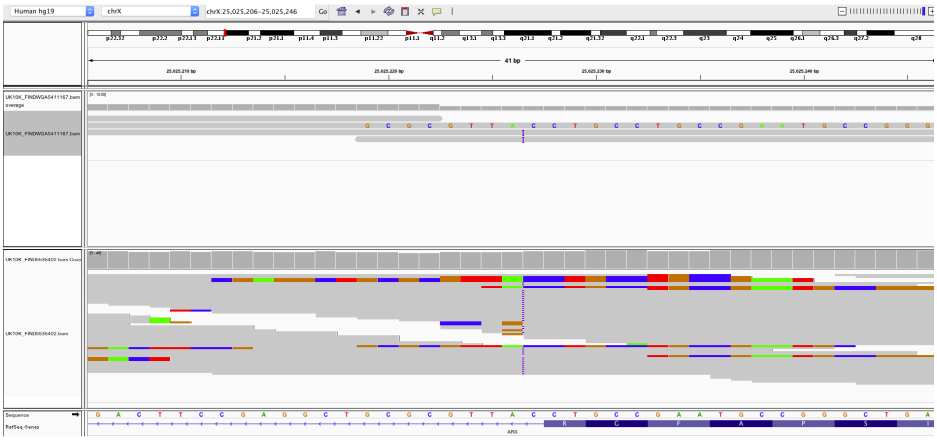

B) F004-A: *UBE3A,* chr15:g.25616333 CATTGT>C; NM_130839.2:c.983_987del

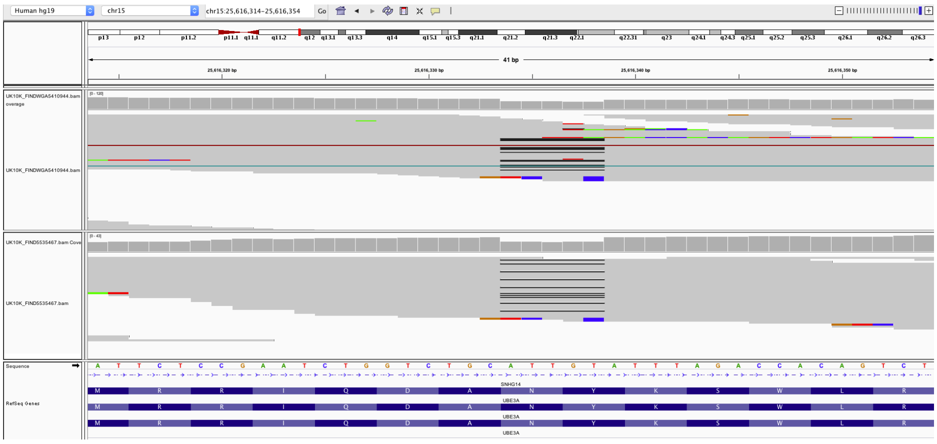

C) F114-A: *SLC6A8,* chrX:g.152960269 C>CG

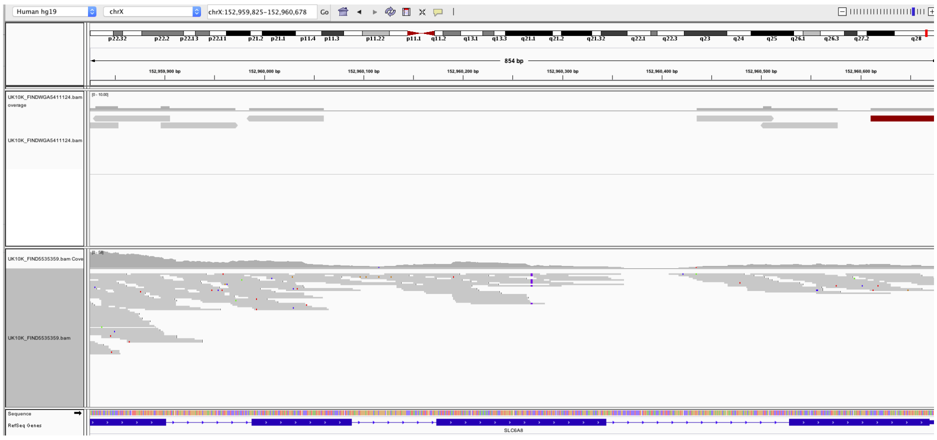

D) F109-B: *ABCD1*, chrX:g.152991575 G>A; NM_000033.3:c.854G>A

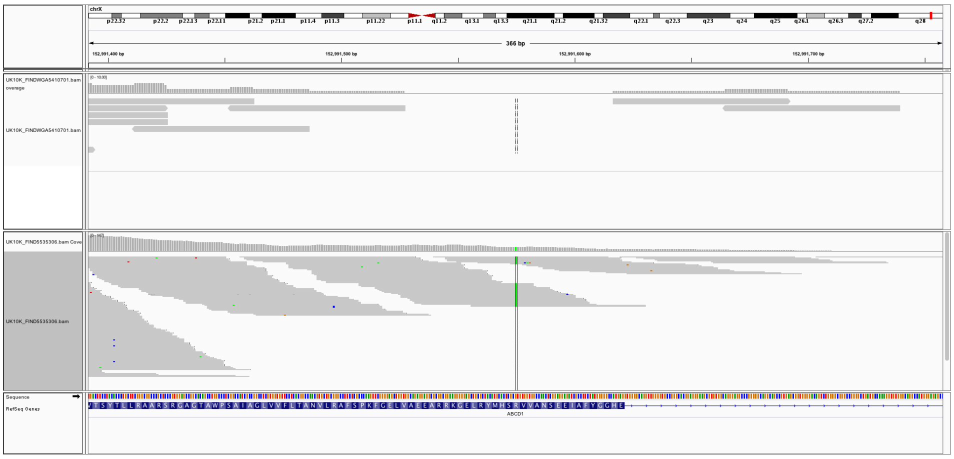

E) F112-A: *GDI1*, chrX:153667457 C>T; NM_001493.2:c.359C>T

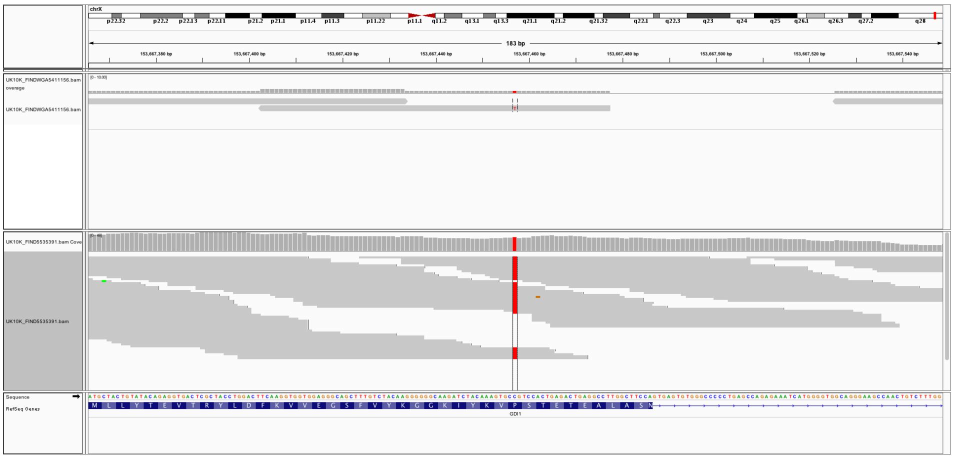

2.4 Figure S4

Figure S4. A) Number of families with reported variants identified in the studied cohort by mode of inheritance. If multiple genes with different mode of inheritance are identified in a family, the most deleterious pathogenicity is considered. **B)** Number of families are grouped if the variants are: shared by all the family members (Same), only present in one individual (One) or multiple family members have different reportable variants (Different). Shading represents the mode of inheritance of the variant/s. AD=autosomal dominant; AR=autosomal recessive; XLR=X-linked recessive.

A)

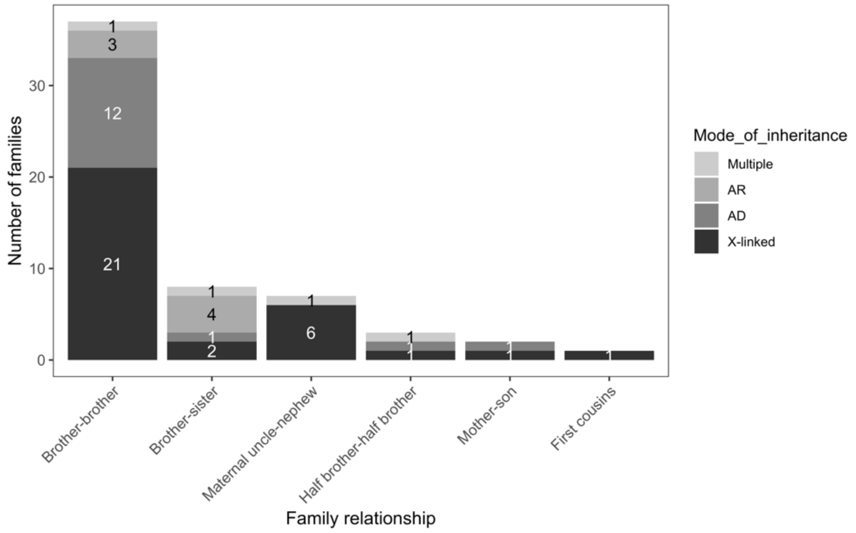

B)

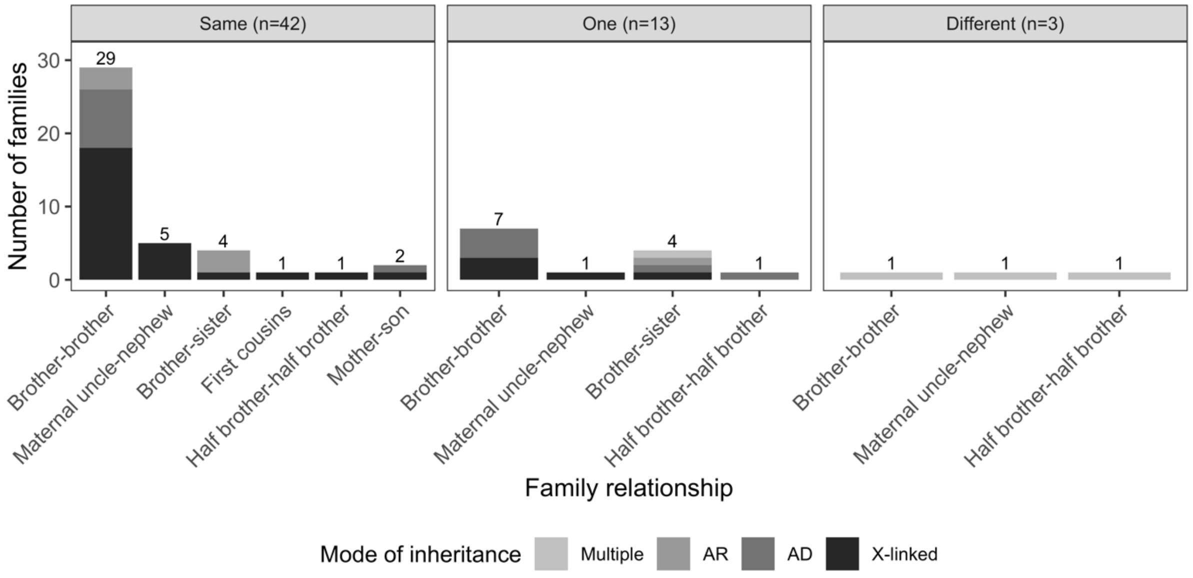

3. References

Ryan L. Collins, Matthew R. Stone, Harrison Brand, Joseph T. Glessner, Michael E.Talkowski. CNView: a visualization and annotation tool for copy number variation from whole-genome sequencing. bioRxiv 049536; doi: https://doi.org/10.1101/049536

Bain JM, Cho MT, Telegrafi A, Wilson A, Brooks S, Botti C, et al. (2016). Variants in HNRNPH2 on the X Chromosome Are Associated with a Neurodevelopmental Disorder in Females. Am J Hum Genet. doi: 10.1016/j.ajhg.2016.06.028.

Robinson JT, Thorvaldsdóttir H, Winckler W, Guttman M, Lander ES, Getz G, Mesirov JP. Integrative genomics viewer. Nat Biotechnol. doi: 10.1038/nbt.1754.

Torti, E., Keren, B., Palmer, E.E., Zhu, Z., Afenjar, A., Anderson, I.J., et al. (2019). Variants in TCF20 in neurodevelopmental disability: description of 27 new patients and review of literature. Genet Med. doi: 10.1038/s41436-019-0454-9.

Bengani, H., Handley, M., Alvi, M., Ibitoye, R., Lees, M., Lynch, S.A., et al. (2017). Clinical and molecular consequences of disease-associated de novo mutations in SATB2. Genet Med 19(8), 900-908. doi: 10.1038/gim.2016.211.
